# The efficacy and safety of sulforaphane as an adjuvant in the treatment of bipolar depressive disorder: Study protocol for a randomized, double-blinded, placebo-controlled, parallel-group clinical trial

**DOI:** 10.1097/MD.0000000000020981

**Published:** 2020-06-26

**Authors:** Congchong Wu, Xingyang Chen, Jianbo Lai, Yi Xu, Shaohua Hu

**Affiliations:** a Department of Psychiatry, The First Affiliated Hospital, College of Medicine, Zhejiang University, Hangzhou; b Taizhou Second People's Hospital, Taizhou; c The Key Laboratory of Mental Disorder Management of Zhejiang Province; d Brain Research Institute of Zhejiang University, Hangzhou, China.

**Keywords:** bipolar disorder, randomized clinical trials, sulforaphane

## Abstract

**Background:**

Bipolar disorder (BD) is a chronic and disabling psychiatric disorder. The treatment of BD still remains a significant clinical challenge due to the complex nature of the disease. Nutraceutical therapy as adjunctive role is a promising therapy for BD. Sulforaphane (SFN), a broccoli extract, was reported to be effective for emotional problems and cognitive impairment. However, clinical research of SFN in the treatment of BD was rare. Therefore, this study is designed to evaluate the adjuvant role of SFN in the treatment of BD.

**Methods:**

This is a randomized, double-blinded, placebo-controlled, parallel-group clinical trial. A total of 100 patients who meet inclusion criteria will be assigned to receive quetiapine plus SFN or quetiapine plus placebo in a 1:1 ratio. The total duration of the study will be 12 weeks including 5 follow ups. The primary outcome is in the Montgomery–Asberg depression rating scale. The secondary outcomes are the quick inventory of depressive symptomatology—self report, Hamilton anxiety rating scale, young mania rating scale, cognitive function, inflammatory factors, and intestinal flora. Any adverse events will be recorded throughout the trial.

**Discussion:**

This trial will provide evidences to evaluate the efficacy and safety of SFN combined with quetiapine in the treatment of BD patients, as well as the adjuvant role of SFN in combination.

**Trial registration:**

This study protocol was registered at the Chinese clinical trial registry (ChiCTR2000028706).

## 1 Introduction

Bipolar disorder (BD) is a common psychiatric disorder characterized by manic and depressive episodes, leading cause of global disability. Bipolar depressive disorder is associated with longer illness duration and poorer response to treatment than mania, impairing quality of life, social relationship, and occupational performance.^[1,2]^ The treatment of bipolar depression is mainly emotional stabilizer, second generation antipsychotics. Clinical recommendation supported atypical antipsychotic drugs as quetiapine, lurasidone for treatment of acute bipolar depression.^[3,4]^ Adverse events (AEs) at recommended doses of the psychoactive agents including akathisia, somnolence, sedation, and metabolic syndrome are associated with treatment compliance and poor treatment outcomes for patient with BD.^[3,5–7]^ Hence, for managing BD, more effective and safer interventions are urgently needed.

There is now significant evidence implicating genetic susceptibility, neurotransmitter imbalance, inflammatory oxidative stress, neurotrophic factor signal transduction deficiency, and neuroendocrine abnormality in the pathophysiology of BD.^[8–13]^ Activated immunity and elevated biomarkers for inflammation were found in patients with BD.^[14,15]^ In addition, compared with the healthy controls, there were inflammatory changes in the prefrontal cortex of BD patients in post-mortem studies, mainly manifested by decreased anti-inflammatory factor level and increased inflammatory factor level.^[16]^ The widely accepted hypothesis of inflammation, oxidative stress, and abnormal immune regulation are therapeutic targets for bipolar depression.

Recently, there has been an increasing interest in the use of plant-derived natural products as complementary treatments with less toxic and fewer side effects. Sulforaphane (1-isothiocyanate-4-methylsulfonyl-butane, SFN), a common antioxidant derived from cruciferous vegetables has been used widely in cancer treatment.^[17]^ It plays a role in the antioxidant and prooxidant activities, canonically acting through the inhibition of inflammatory cytokine production and down regulation of nuclear factor-kappa B activity which ultimately affect the occurrence and progression of mental disorders.^[18–21]^ At present, SFN has been used to treat mental disorder in the clinical studies, such as autism, and has achieved positive results.^[22,23]^ Shiina et al reported that SFN improved cognitive function in patients with schizophrenia.^[24]^ What's more, SFN was proving owning a certain inhibitory effect on depression-like behavior associated with inflammation-induced depression in mice.^[25–27]^ However, the adjuvant effect of SFN in the treatment of patients with BD has never been studied and remains unknown.

In summary, SFN can influence the oxidative stress–inflammation pathways, resulting in a positive effect on cognitive and emotional development. This study aims to observe the adjuvant effects or improvement effects and safety of SFN in the quetiapine for bipolar depressive disorder, and explore the mechanisms by detecting changes in inflammatory factors and intestinal flora before and after treatment.

## 2 Methods

### 2.1 Study design and ethics approval

**Figure 1:**
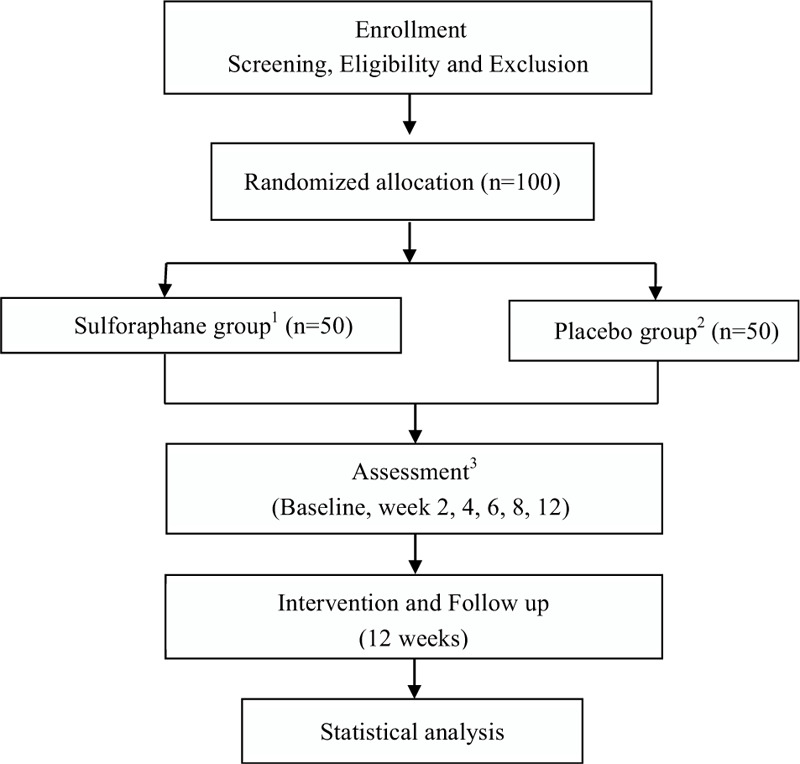
Flow chart of the study.

This is a randomized, double-blinded, placebo-controlled, parallel-group clinical trial. This protocol was approved by the Research Ethics Committee of the First Affiliated Hospital, College of Medicine, Zhejiang University (December 24th, 2019). The trial which is registered at the Chinese Clinical Registry (ChiCTR2000028706) will be conducted in the First Affiliated Hospital, College of Medicine, Zhejiang University in China. The clinic trial was supported by the grant of the National Natural Science Foundation of China (81971271). Figure 1 depicts a flow chart of the study. Patients enrolled in this study will randomly be assigned into 2 groups (with a 1:1 allocation ratio). Clinical manifestations and assessment scales, neurocognitive functions, and some of the blood biochemical tests will be checked before treatments started (baseline) and will be reexamined on week 2, 4, 8, 12 weeks of treatment to assess the effectiveness and safety. Meanwhile, inflammatory cytokines and intestinal flora were monitored during follow-up. Researchers will record and deal with the any adverse reactions and assess whether they can continue the experiment to protect the participants’ interests during the study. Specific schedules are summarized in Table 1.

**Table 1:**
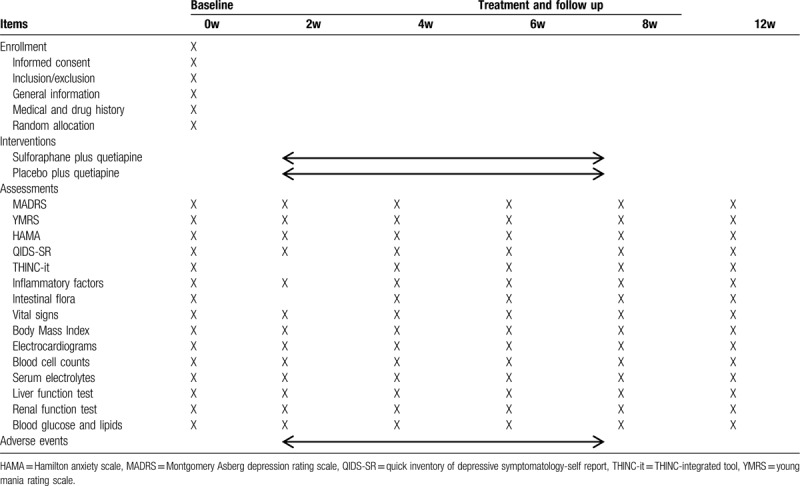
Schedule of the enrollment, intervention, and assessments in the process.

### 2.2 Study population

Participants who meet the eligibility criteria and sign informed consent will be enrolled in the study. Inclusion criteria for enrollment are described as follows:

(1) males or females aged 16 to 65 years old;
(2) agree to participate in the research and sign the informed consent form;
(3) the subjects biological parents are Han;
(4) diagnosed with bipolar depressive disorder according to the fourth Edition of Diagnostic and Statistical Manual of Mental Disorders diagnostic criteria;
(5) score more than or equal to 17 points on the Hamilton depression scale at screening and baseline period;
(6) score less than or equal to 5 points on the young mania rating scale (YMRS) at screening and baseline period;
(7) no taking any drug treatment or no receiving antidepressant treatment in the last 1 month;
(8) take drugs by oneself during the study period, or have a regular helper to help with the medication.

Exclusion criteria are described as follows:

(1) diagnosed with other spectrum disorders according to the fourth Edition of Diagnostic and Statistical Manual of Mental Disorders diagnostic criteria;
(2) mental disorder caused by substance abuse, or serious physical diseases;
(3) received antibiotics within 1 month before enrollment due to respiratory tract, urinary tract, the digestive system infection;
(4) subjects with a history of attempted suicide, or currently at high suicide risk, or with suicide behavior/attempt, or scoring more than or equal to 3 points on the 10th clause of Montgomery Asberg Depression Rating Scale (MADRS);
(5) unwilling to take the medicine;
(6) known pregnancy, lactation, or pregnant planning;
(7) the subjects with contraindications of quetiapine or have used quetiapine but have poor efficacy;
(8) allergic to broccoli;
(9) received electroconvulsive therapy within 1 month.

Suspension criteria are as follows:

(1) subjects with serious adverse drug reactions;
(2) exchange drugs or receive other treatment due to unstable condition;
(3) refuse to continue participating in the study;
(4) lose track in follow-up;
(5) other factors cause the interview to stop.

### 2.3 Randomization and blinding

The enrolled participates will be randomly assigned a random number sequence which generated by SAS ver.9.0 for Microsoft Windows (SAS InstituteInc, Cary, NC) in a 1:1 ratio. A sealed envelope, independently managed by a third researcher was used to allocation concealment. The researcher will not take part in follow-up and assessment. Neither assessors nor subjects will know the assigned group and the intervening measure until the study is terminated.

### 2.4 Groups and interventions

In this study, enrolled bipolar depressed patients are randomly assigned into 2 groups: SFN group and placebo group and will undergo 12-week quetiapine concomitantly. The initial treatment dose of quetiapine in the 2 groups are 50 mg daily, and will be added to 300 mg daily within 2 weeks. The patients in SFN group will receive 1 tablet of Avmacol Extra Strength (480 mg) daily swallowed with warm water (no more than 40 degrees) after breakfast or chewed at the same meal, and placebo group will receive a placebo in the same way. Avmacol Extra Strength contains Proprietary Sulforaphane Glucosinolate (Glucoraphanin) & Myrosinase Blend 490 mg including Broccoli Seed & Sprout Extract (containing more than 30 mg of glucoraphanin). The placebo matches Avmacol Extra Strength with size, shape, taste, and package without any active ingredient. Both of them used in this study are produced by the Nutramax Laboratories Consumer Care (Lancaster, SC, USA).

### 2.5 Outcomes

Primary outcome is the change in the MADRS scores at baseline compared to each treatment time point. MADRS is a clinician-administered rating scale and widely used to assess the depression severity of patient with BD. The response was defined as at least a 50% reduction when compared to baseline in the scores.

While secondary outcomes include the scores of the quick inventory of depressive symptomatology-self report, Hamilton anxiety rating scale, and YMRS scores are used to elevate the broader affective symptoms. Besides, cognitive function which tested by THINC-integrated tool, inflammatory factors (interleukin-2, interleukin-4, interleukin-6, interleukin-10, interleukin-17A, tumor necrosis factor and interferon-γ) and intestinal flora are used to investigate the underlying biological factors.

### 2.6 Safety assessment

Vital signs, body mass index, the liver and kidney function, levels of serum electrolytes, blood glucose and lipids, blood cell counts, and electrocardiograms will be conducted on all participants. At each participant's visit, AEs will be assessed. The severity of AEs were assign as mild, moderate, severe, or serious. We will record the onset date, end date, severity, the relationship with the clinical trial, the outcomes, and the measures adopted in the AEs form and provide appropriate treatment if any AEs occur during the study process. The trial will be stopped in case of treatment-related suspected unexpected serious AEs.

### 2.7 Sample size

It was absent of direct evidences to support t our hypothesis, consequently, estimating the sample size is difficult. At an 8-week study, the response rate of quetiapine in BD patients was 58.9%, and the remission rate was 42.1%.^[28]^ Combination therapy has been shown to be more effective. We estimated the effective rate of SFN combined with quetiapine in the treatment of bipolar depressive disorder to be about 70%. The sample size with 80% power at a significance level of 0.05 was calculated in this study. Anticipating a 10% dropout rate, a total of 100 patients, with 50 patients in each group, will be needed.

### 2.8 Statistical analysis

All statistical analyses will be performed using SPSS 19.0 software (IBM, Armonk, NY) in a blinded manner. Outcomes will be analyzed on the basis of intention-to-treat principle. Missing values will be handled by the practical guide described by Jakobsen et al. The comparison of primary outcomes and secondary outcomes between 2 groups will be performed by *t* test for continuous variables and *χ*^2^ test for categorical variables, or with Wilcoxon Mann–Whitney test. The paired *t* test will be used for analysis in each group. *P* < .05 will be considered significant

## 3 Discussion

As a chronic and complex mood disorder, the treatment of BD frequently required combinations of therapies. A number of pharmacotherapeutic treatments with side effects induce low adherence of patient and poor outcomes. Nutraceutical agents with higher acceptability are more and more considered to improve treatment for the symptoms of BD as adjunctive therapies.^[29]^ In the treating mental disorders, SFN has been proved have a significant improvement in mood and cognitive function by regulating peroxidation reactions.^[25,30]^ The aim of this randomized clinical trial is to investigate the effect of SFN as an adjuvant role in the treatment of bipolar depressive disorder.

In this study, besides using the MADRS, Hamilton depression scale, Hamilton anxiety rating scale, quick inventory of depressive symptomatology-self report, and YMRS scales to assess the clinical curative effect on emotional problem, THINC-integrated tool will be introduced to evaluate neurological status. In addition, we monitor inflammatory factors including serum concentration of and intestinal flora before and after treatment to explore the possible mechanism of SFN combined with quetiapine on treating bipolar depressive disorder. In summary, we expected that our findings will provide evidence for efficacy and safety of SFN as an adjuvant treatment of BD.

This study also has some limitations. First, this is an exploratory trial with evaluating the intervention by various outcomes and process measures from multiple perspectives, the results should be cautiously interpreted. Besides, as a nutritional supplement, the dose of SFN in the preliminary study is conventional, but perhaps that's not necessarily a most effective dose. Thus, further study is required to explore an optimal dose plan for SFN as an adjuvant in the treatment of bipolar depressive disorder.

In summary, this trial is expected to provide initial findings on the synergistic effect of SFN in the treatment of BD. If, as expected, combination of quetiapine and SFN therapy could have more positive effects, it will provide an opportunity to increase the availability of evidence treatment for patients with BD.

## Author contributions

**Conceptualization:** Xingyang Chen.

**Supervision:** Shaohua Hu, Yi Xu.

**Writing – original draft:** Congchong Wu.

**Writing – review & editing:** Jianbo Lai.
